# Human HLA prolongs the host inflammatory response in *Streptococcus suis* serotype 2 infection compared to mouse H2 molecules

**DOI:** 10.3389/fcimb.2023.1285055

**Published:** 2023-11-14

**Authors:** Chengpei Ni, Yi Han, Yajing Wang, Ting Ma, Dan Sha, Yanan Xu, Wenting Cao, Song Gao

**Affiliations:** ^1^The Affiliated Wuxi Center for Disease Control and Prevention of Nanjing Medical University, Wuxi Center for Disease Control and Prevention, Wuxi, China; ^2^School of Public Health, Nanjing Medical University, Nanjing, China; ^3^State Key Laboratory of Pathogen and Biosecurity, Institute of Microbiology and Epidemiology, Academy of Military Medical Sciences, Beijing, China; ^4^Wuxi Medical Center, Nanjing Medical University, Wuxi, China

**Keywords:** *Streptococcus suis*, humanized transgenic mouse, MHC, inflammation, immunoregulation

## Abstract

*Streptococcus suis* (*S. suis*) is widely acknowledged as a significant zoonotic pathogen in Southeast Asia and China, which has led to a substantial number of fatalities in both swine and humans. Despite the prevalent use of mice as the primary animal model to study *S. suis* pathogenesis, the substantial differences in the major histocompatibility complex (MHC) between humans and mice underscore the ongoing exploration for a more suitable and effective animal model. In this study, humanized transgenic HLA-A11/DR1 genotypes mice were used to evaluate the differences between humanized HLA and murine H2 in *S. suis* infection. Following intravenous administration of *S. suis* suspensions, we investigated bacterial load, cytokine profiles, pathological alterations, and immune cell recruitment in both Wild-type (WT) and humanized mice across different post-infection time points. Relative to WT mice, humanized mice exhibited heightened pro-inflammatory cytokines, exacerbated tissue damage, increased granulocyte recruitment with impaired resolution, notably more pronounced during the late infection stage. Additionally, our examination of bacterial clearance rates suggests that HLA-A11/DR1 primarily influences cell recruitment and mitochondrial reactive oxygen species (ROS) production, which affects the bacterial killing capacity of macrophages in the late stage of infection. The reduced IL-10 production and lower levels of regulatory T cells in humanized mice could underlie their compromised resolution ability. Intervention with IL-10 promotes bacterial clearance and inflammatory regression in the late stages of infection in transgenic mice. Our findings underscore the heightened sensitivity of HLA-A11/DR1 mice with impaired resolution to *S. suis* infection, effectively mirroring the immune response seen in humans during infection. The humanized HLA-A11/DR1 mice could serve as an optimal animal model for investigating the pathogenic and therapeutic mechanisms associated with sepsis and other infectious diseases.

## 1 Introduction

Strains of serotype 2 *Streptococcus suis* (*S. suis*), as zoonotic pathogens, pose a significant threat to public health and lead to substantial economic losses in the swine industry, particularly in Asian countries (Ni et al., 2023). The bacterial transmission occurs from diseased swine, the primary epidemic source, to individuals in close contact with infected pigs or pork meat. This transmission results in various symptoms, including bacteremia, streptococcal toxic shock-like syndrome (STSLS), and meningitis. A common clinical feature of *S. suis* infections is severe bacteremia, indicating the presence of viable bacteria in the bloodstream of patients. Notably, the infection is prevalent in Northern Europe and Southeast Asia, with recent reports of two massive outbreaks in China.

The impact of *S. suis* varies significantly not only between different nations but also within individual countries. The pathogenicity of distinct strains varies markedly across countries and continents. While a large number of deaths due to *S. suis* infections have been reported in Asia, most *S. suis* strains reported in North America are non-virulent and lack pathogenicity (Gottschalk et al., 2007; Zouharova et al., 2022). This phenomenon can be attributed to the presence of diverse endemic strains carrying varying virulence factors in different regions. For instance, the majority of epidemic *S. suis* serotype 2 strains in China carry an 89K pathogenicity island (PAI), which is closely associated with large-scale outbreaks (Li et al., 2011; Wang et al., 2022). However, this PAI is rarely found in European or North American strains. Furthermore, the diversity of major histocompatibility complex (MHC) molecular polymorphisms in different human populations is likely another contributing factor to the differences in pathogenicity among *S. suis* strains. MHC restriction exhibits marked differences based on geographical regions and ethnicities, with over 15,000 distinct classical HLA class I and II alleles identified. This diversity is strongly linked to a wide array of human diseases, including autoimmune and infectious diseases (Radwan et al., 2020; Taylor and Balko, 2022).

Undoubtedly, pigs are the most suitable animals for studying *S. suis* pathogenesis due to their relevance, but their size, space requirements, and high cost limit their use in research. In contrast, mice possess attributes that make them desirable for modeling (Parker, 2017). However, it is important to note that the dose of *S. suis* required to induce infection in mice far exceeds that used in pigs or humans. Murine models of *S. suis* infection suffer from high infectious doses and poor bacterial viability in the blood (Neila-Ibanez et al., 2021). The virulence of *S. suis* serotype 2 varies between mice and pigs due to host specificity. The rise of emerging infectious diseases necessitates new pre-clinical animal models, prompting the need for more suitable and optimized disease models.

Humanized mouse models, where mice express human MHC-I/II molecules, effectively mimic human antigen processing and presentation machinery. These models are widely used for vaccine evaluation and reproducing idiosyncratic drug toxicity (Zhu et al., 2022). Studies have shown that Tg HLA molecules are recognized by the mouse immune system akin to alternate mouse H2 class I alleles (Cheuk et al., 2002). However, their application in bacterial infections, especially those lacking superantigens, is limited. In this study, we employed an HLA-A11/DR1 transgenic (Tg) mouse model (based on the HLA-A11/DR1 genotypes), which represents 10–15% of the Chinese population (Zeng et al., 2016; He et al., 2018; Li et al., 2019). This model was used to assess differences in antigen processing, inflammatory responses, and bacterial clearance between humanized HLA molecules and murine H2 molecules (Zeng et al., 2016; Li et al., 2019). The aim of this study was to comprehensively examine the role of human HLA molecules during *S. suis* infection using this humanized Tg mouse model, evaluating immune responses against *S. suis* strains isolated from patients during a Chinese outbreak.

## 2 Materials and methods

### 2.1 Mice and ethics statement

Female humanized transgenic C57BL/6 mice HLA-A11/DR1 (HLA-A11^+/+^DR1^+/+^H-2- $\beta_{2m}^{-/-}$ /IA*β*^-/-^), aged 6-8 weeks, were generously provided by Dr. Yusen Zhou of the Academy of Military Medical Sciences, Beijing, China. Information about these Tg mice was previously published (Zeng et al., 2016). In brief, homozygous HLA-A11/DR1 Tg mouse was generated by crossing HLA-A11 transgenic (Tg) mice with HLA-A2^+/+^DR1^+/+^H-2-- $\beta_{2m}^{-/-}$ /IA*β*^-/-^ mice. HLA-A11 Tg mice were bred to express an MHC molecule incorporating the α1, α2, and *β*_2_m domains of human HLA-A11, along with the α3 transmembrane and cytoplasmic domains of murine H-2Db. Wild-type (WT) female C57/BL6J mice (HLA-A11^-/-^DR1^-/-^H-2- $\beta_{2m}^{+/+}$ /IAb^+/+^), 6–8 weeks old, were obtained from SPF (Beijing, China) Biotechnology Co., Ltd and served as controls. Mice were acclimatized under standard laboratory conditions.

### 2.2 Bacterial strains

The *S. suis* serotype 2 strain 05ZYH33 employed in this research was derived from the clinical isolates during the Chinese *S. suis* pandemic in 2005. This virulent strain, encapsulated, suilysin-positive, and 89K PAI-positive, was utilized for all experimental infections. *S. suis* was cultured in Todd-Hewitt broth (THB) medium at 37°C, and bacteria in the mid-log growth phase were harvested for experimentation.

### 2.3 Infection

For infection, 100 µl of either the bacterial suspension (1 x 10^8^ CFU per mouse) or the vehicle solution (THB) was administered intravenously (i.v.). Infected mice were vigilantly monitored daily to record mortality, bacterial load, clinical signs of disease, and weight loss associated with infection. For Scoring, all mice were observed for a duration of 7 days, and clinical scores were allocated based on established criteria from a prior study (Lin et al., 2019): 0 denoting a normal response to stimuli, 1 indicating a ruffled coat and sluggish response to stimuli, 2 representing response solely to repetitive stimuli, 3 signifying non-responsiveness or circular ambulation, 4 indicating fatality. In the intervention experiment, HLA-A11/DR1 mice were subjected to random allocation for the administration of 1 μg of murine IL-10 (PeproTech, USA). This recombinant cytokine was reconstituted in a 1% homologous mouse serum solution, prepared in phosphate-buffered saline (PBS). The control group was injected with PBS containing 1% homologous mouse serum solution. The administration was accomplished via intraperitoneal injection, 48 h following the induction of *S. suis* infection (Kaufman et al., 1999). Subsequently, bacterial loads at 72 h and 96 h post-infection, cytokine levels, and histological changes at 72 h post-infection were assessed.

### 2.4 Flow cytometry

At each indicated time point, mice were euthanized, and spleens were collected. After fragmentation, filtration, and milling, single cells were obtained. ACK Lysis Buffer was employed to selectively lyse erythrocytes, leaving the leukocytes intact. FcR was blocked using CD32/CD16 antibodies (S17011E). Cells were stained individually following this protocol: Panel 1: FITC-anti-mouse CD45 (30-F11), PE-anti-mouse NK1.1 (PK136), PerCP-Cy5.5-anti-mouse CD4 (GK1.5), PE-Cy7-anti-mouse B220 (RA3-6B2), APC-anti-mouse CD3 (145-2C11), APC-Cy7-anti-mouse CD8a (53-6.7). Panel 2: FITC-anti-mouse CD45 (30-F11), PE-anti-mouse Ly6G (RB6-8C5), PerCP-Cy5.5-anti-mouse CD11b (M1/70), PE-Cy7-anti-mouse Ly6C (HK1.4), APC-anti-mouse F4/80 (BM8), APC-Cy7-anti-mouse fixable viability dyeeFluor780 (eBioscience). Panel 3: FITC-anti-mouse CD45 (30-F11), PE-anti-mouse CD11c (N418), PerCP-Cy5.5-anti-mouse CD11b (M1/70), PE-Cy7-anti-mouse NK1.1 (PK136), APC-Cy7-anti-mouse fixable viability dyeeFluor780. The frequency of regulatory T Cells (Tregs) (Foxp3^+^ regulatory CD4 T cells) in splenocytes was analyzed using the anti-mouse Foxp3 staining kit from Invitrogen (San Diego, CA, USA), following the manufacturer’s protocol. Flow cytometric analysis of leukocyte subsets was performed using a BD Verse (BD Biosciences, USA) equipped with a six-laser system.

### 2.5 Quantification of cytokines

Plasma cytokine levels were measured through ProcartaPlex™ Multiplex Immunoassay (Invitrogen, USA), following the manufacturer’s instructions. The measured cytokines included GM-CSF, IFN-γ, IL-1 β, IL-12p70, IL-13, IL-18, IL-2, IL-4, IL-5, IL-6, TNF-α, ENA78, G-CSF, IFN-α, IL-1 α, IL-15R, IL-28, IL-3, IL-31, LIF, M-CSF, IL-10, IL-17A, IL-22, IL-23, IL-27, IL-9, Eotaxin, GRO-α, IP-10, MCP-1, MCP-3, MIP-1a, MIP-1b, MIP-2, RANTES. In some experiments, the concentrations of IL-6, INF-γ, and TNF-α in the plasma mice were determined using enzyme-linked immunosorbent assay (ELISA) kits (Neobioscience, China) according to the manufacturer’s instructions.

### 2.6 Histology

Formalin-fixed tissues underwent a process of dehydration, embedding, sectioning, and subsequent utilization for hematoxylin-eosin staining (H&E). Subsequently examined for histopathological changes using an Olympus BX-53 microscope.

### 2.7 Isolation of neutrophils

Cell isolation and characterization were executed in accordance with previous protocols (Swamydas et al., 2015). Briefly, donor mice were intraperitoneally administered with 1.0 ml of casein solution, followed by an injection after an overnight interval. Peritoneal fluid was collected three hours following the second injection. The collected peritoneal exudate cells were then mixed with a 9 ml volume of Percoll gradient solution within a 10 ml ultracentrifuge tube. After undergoing ultracentrifugation for 20 min at 60,650 × g in 4°C. Discard the thin upper layer, neutrophils located within the second distinct opaque layer were selectively harvested. After washing the cells with PBS 3 times, the assessment of cell purity and viability was performed using the trypan blue dye exclusion and FACS methodology.

### 2.8 Luminol-enhanced chemiluminescence

Reactive oxygen species (ROS) were quantified using luminol-enhanced chemiluminescence (Barnes et al., 2012). Neutrophils (5×10^4^ cells) were added to a non-coated 96-well microplate. Luminol (5-amine-2,3-dihydro-1,4-phtalazinedione) was added at a final concentration of 67 µM, neutrophils alone served as the negative control. Chemiluminescence was immediately measured using a PerkinElmer microplate reader at 3-mins intervals for 20 cycles.

### 2.9 Evaluation of mitochondrial ROS *in vivo*

Splenocyte cell suspensions from mice in 72 h post-infection were collected. To selectively characterize monocytes/macrophages, an acutely isolated monocytes/macrophages fraction was obtained using MACS microbeads (Miltenyi Biotec) conjugated with CD11b antibody. For the *in vivo* assessment of mitochondrial ROS generation in macrophages and monocytes, cells were plated in a 96-well plate at a concentration of 1 × 10^6^ cells per ml, with each well containing 100 µl of the cell suspension. Subsequently, 5 µM of MitoSOX was introduced to each well. MitoSOX is a mitochondria-specific dye that fluoresces red upon oxidation (Kim et al., 2009). The fluorescence resulting from the oxidation of MitoSOX was measured using a Molecular Devices SpectraMax i3 plate reader, employing excitation at 510 nm and emission detection at 580 nm, following cell activation (Azzouz and Palaniyar, 2023).

### 2.10 Bacterial clearance function in macrophage

Bone marrow-derived macrophages (BMDM) were obtained following a previously established protocol, with minor adjustments as documented in reference (Toda et al., 2021). Bone marrow cells were harvested from mice of both genotypes, followed by centrifugation at 200 × g for 5 minutes at 4°C, and subsequent aspiration of PBS. After thorough washing, filtration, and erythrocyte lysis, the cell suspension was transferred into the bone marrow culture medium, comprising DMEM with 10% FBS, 1% penicillin/streptomycin, and 10 ng/mL of macrophage colony-stimulating factor (M-CSF). These cells were cultured under standard conditions at 37°C with 5% CO_2_. To obtain differentiated macrophages, 5 ng/ml of macrophage colony-stimulating factor was added every 2 d for 6 d. Adherent cells were collected on day 7 of culture as BMDM. Before conducting experiments, BMDM were transitioned to an antibiotic-free culture medium and allowed to incubate overnight. For bacterial infection, BMDM were exposed to *S. suis* at a multiplicity of infection (MOI) of 10. After 60 minutes of infection, gentamicin treatment was administered to eliminate extracellular bacteria. Subsequently, the number of live bacteria was quantified by plating on THY agar supplemented with ampicillin (50 μg/ml) at 1 h intervals (for phagocytosis) or 6 h intervals (for bacterial killing) post-infection (Kang et al., 2020). In the context of the mitochondrial ROS assay, cells were seeded in a 96-well plate at a concentration of 1 × 10^6^ cells per ml, with each well receiving 100 µl of the cell suspension. To assess mitochondrial ROS, 5 µM of MitoSOX was introduced to each well. The encounter between cells and *S. suis* at a MOI of 20 occurred at the indicated time points. The oxidation of MitoSOX was quantified using a Molecular Devices SpectraMax i3 plate reader.

### 2.11 Statistics

Bacterial loads in different tissues and plasma cytokine levels between C57/BL6J and HLA-A11/DR1 mice were compared using the Mann-Whitney test. The obtained data exhibited normal distribution, and differences in means of Luminol-enhanced chemiluminescence, mitochondrial ROS, phagocytosis, and bacterial killing were analyzed using the paired Student’s t-test. All data analysis and statistics in this study were conducted using GraphPad Prism. *P* value < 0.05 was considered significant.

## 3 Results

### 3.1 Human HLA-A11/DR1 prolongs bacterial clearance time in *Streptococcus suis* infection

**Figure 1:**
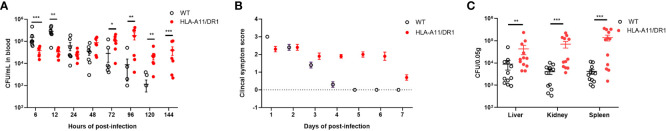
Impact of human HLA-A11/DR1 on *S. suis* infection outcomes. Wild-type (WT) and HLA-A11/DR1 mice were intravenously inoculated with 1.0 × 10^8^ CFU of *S. suis* 05ZYH33. **(A)** Bacterial loads in the blood were assessed at indicated time points (n=7). **(B)** Clinical scores were assigned following defined criteria (n=10). **(C)** Tissue homogenates of the liver, kidney, and spleen at 72 h were evaluated via colony plate counting (n=12). Data are represented as mean ± SEM. *P < 0.05; **P < 0.01; ***P < 0.001.

To investigate whether humanized Tg mice exhibit altered bacterial clearance *in vivo*, WT and humanized HLA-A11/DR1 mice were inoculated with *S. suis* 05ZYH33 (1×10^8^ CFU) through tail intravenous injection, the bacterial loads and clinical manifestation in both genotypes mice were carefully recorded within 144 h post-infection. In comparison to WT mice, significantly lower bacterial loads were observed in the blood of HLA-A11/DR1 mice in 6 h and 12 h, while after 48 h bacterial loads became increased significantly in HLA-A11/DR1 mice (**Figure 1A**). Particularly at 144 h post-infection, the WT group displayed almost no bacteria in the blood, while a substantial number of bacteria were still present in the blood of most HLA-A11/DR1 mice, which suggests that HLA prolongs the survival of bacteria in the blood and affects their clearance by the host immune system. This observation correlated with the clinical scores, indicating more severe clinical manifestations in HLA-A11/DR1 mice during the late stage of infection (**Figure 1B**). As *S. suis*-induced septicemia often leads to organ failure, bacterial loads were also monitored in organs (liver, kidney, and spleen) at 72 h post-infection, when the blood bacterial load of HLA-A11/DR1 started to be significantly higher than that of WT group. Similar to the blood bacterial load results, increased bacterial loads were evident in all organs of HLA-A11/DR1 mice (**Figure 1C**).

These findings suggest that HLA-A11/DR1 impairs bacterial clearance and exacerbates clinical symptoms, particularly in the advanced stage of *S. suis* infection.

### 3.2 Human HLA-A11/DR aggravates the host response to *Streptococcus suis* sepsis

**Figure 2:**
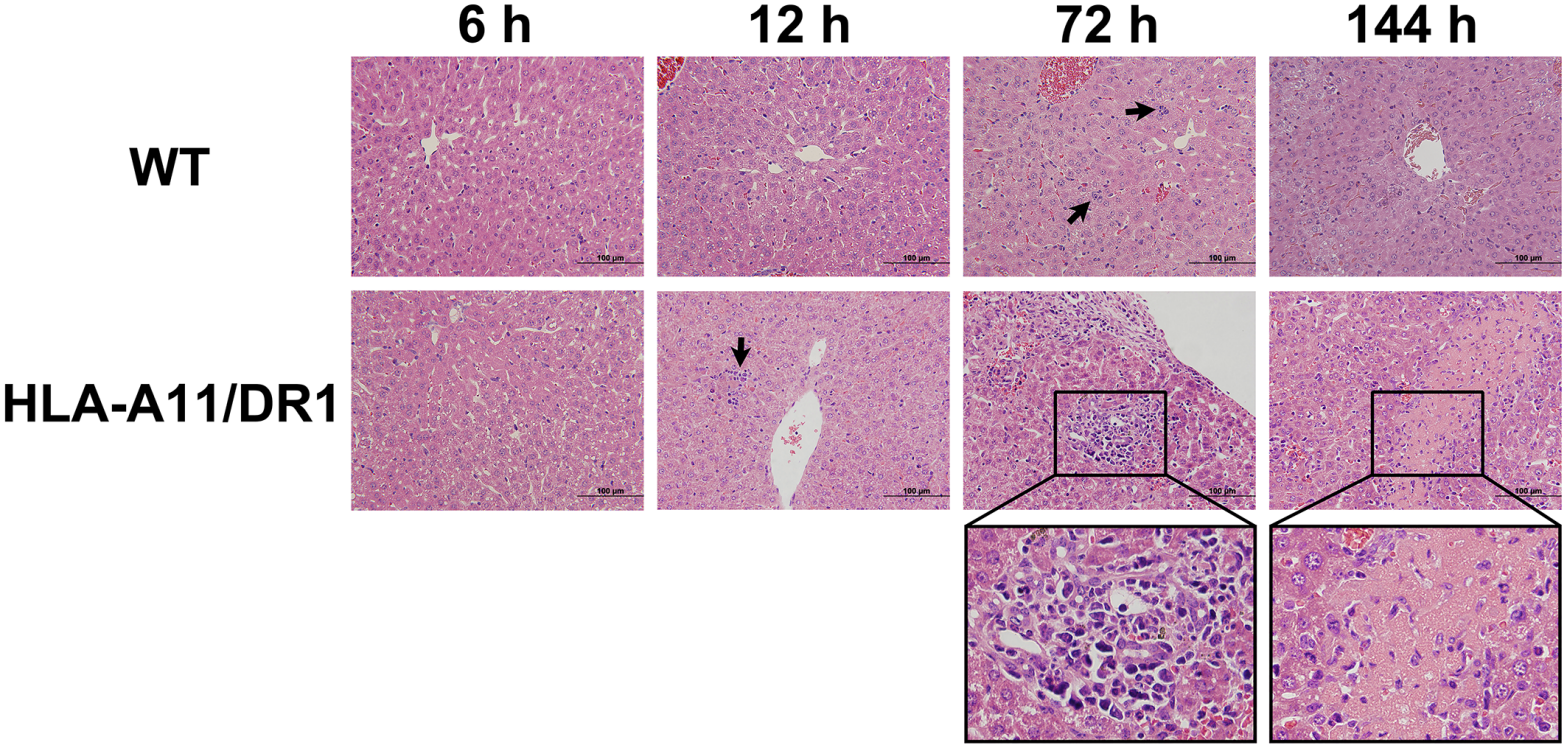
Histopathological analysis of infected liver sections. Hematoxylin and eosin (H&E) staining of infected liver sections from WT and HLA-A11/DR1 mice at 6 h, 12 h, 72 h, and 144 h post-*S. suis* infection. Inflammatory cell infiltration is indicated by a “black arrow”, and liver tissue necrosis is magnified by the “black wireframe”. Original magnification ×100.

Inflammation constitutes the predominant clinical manifestation during *S. suis* infection, often resulting in liver lesions. Thus, livers and plasma from WT and HLA-A11/DR1 mice during infection were collected for pathological examination and cytokine analysis. The role of HLA-A11/DR1 in modulating inflammation during *S. suis* infection was explored in depth. Histologically, infected HLA-A11/DR1 mice exhibited greater necrotic cell presence in the liver compared to WT mice during the late stage of infection (**Figure 2**). Particularly at 72 h and 144 h, clusters of hepatocytes were replaced by inflammatory cells. This significant tissue damage in the advanced infection stage suggested a prolonged and intense inflammatory response in HLA-A11/DR1 mice, consistent with bacterial load monitoring findings.

**Figure 3:**
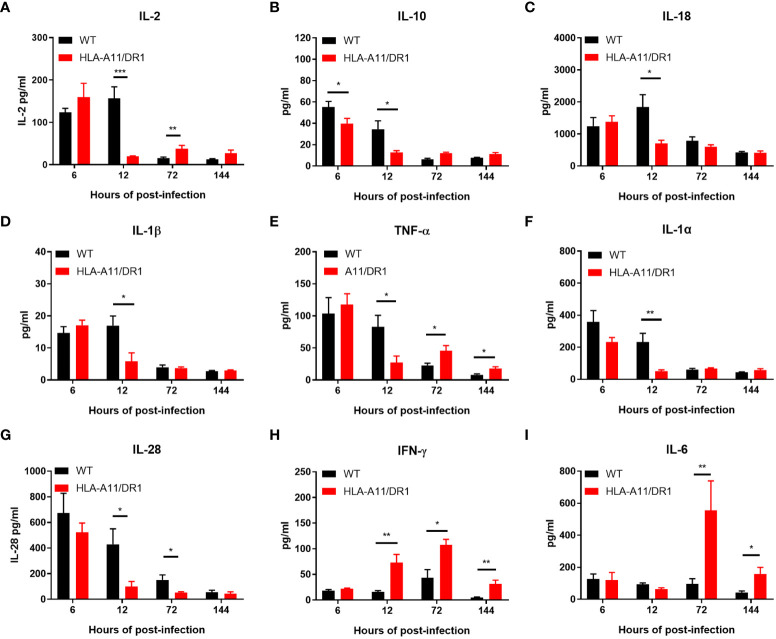
Inflammatory cytokine kinetics in response to *S. suis* infection. Kinetics of inflammatory cytokine expression in WT and HLA-A11/DR1 mice infected intravenously with *S. suis*. Concentrations of **(A)** IL-2, **(B)** IL-10, **(C)** IL-18, **(D)** IL-1β, **(E)** TNF-α, **(F)** IL-1α, **(G)** IL-28, **(H)** IFN-γ, and **(I)** IL-6 in plasma were assessed using Luminex (n=5). Data are represented as mean ± SEM (pg/ml). *P < 0.05; **P < 0.01; ***P < 0.001.

**Figure 4:**
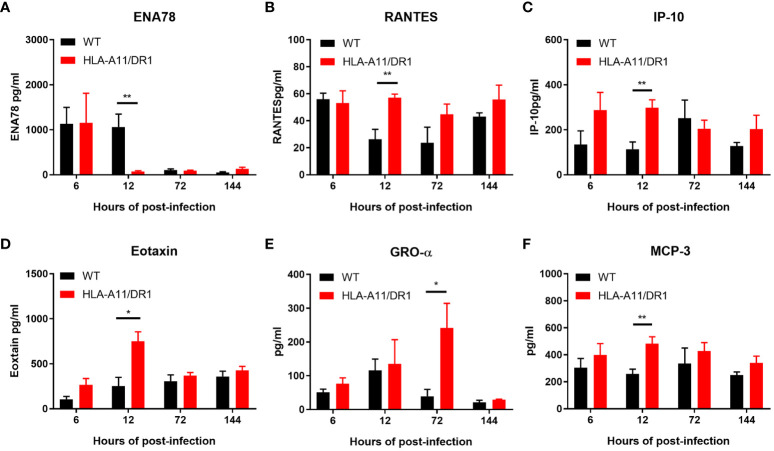
Chemokine kinetics for the reaction to *S. suis* infection. Kinetics of chemokine expression in WT and HLA-A11/DR1 mice infected intravenously with *S. suis*. Concentrations of **(A)** ENA78, **(B)** RANTES, **(C)** IP-10, **(D)** Eotaxin, **(E)** GRO-α, and **(F)** MCP-3 in plasma were determined using Luminex (n=5). Data are represented as mean ± SEM (pg/ml). *P < 0.05; **P < 0.01.

The analysis of plasma using the ProcartaPlex™ Multiplex immunoassay revealed notable differences in the kinetics of inflammatory mediator production between the two genotypes during infection. The HLA-A11/DR1 mice strain induced lower levels of several cytokines (IL-2, IL-10, IL-18, IL-1β, TNF-α, IL-1α, IL-28, and ENA78), but showed increased secretion of IFN-γ, RANTES, IP10, and Eotaxin at 12 h post-infection, compared to the WT group (**Figures 3A–H**, **4A–D**). The infection induced an enduring high inflammatory cytokine response in HLA-A11/DR1 mice after 72 h, including elevated TNF-α, IL-6, IFN-γ, and GRO-α levels compared to the WT mice (**Figures 3E, H, I**, **4E**). These crucial mediators of septic shock exhibited a slow decline and remained at high levels even at 144 h in HLA-A11/DR1 mice. The humanized mice displayed a significant 5-fold increase in IL-6 gene expression compared to the WT group at 72 h (**Figure 3I**). MCP3 was identify as an important chemokine which attract macrophages and monocytes to further amplify inflammatory processes and contribute to disease progression, in our study a higher level of MCP-3 was significantly observed in HLA-A11/DR1 group at 12 h post-infection (**Figure 4F**). Collectively, these findings suggest that humanized HLA-A11/DR1 mice exhibit heightened production of pro-inflammatory cytokines and a more rapid immune response during *S. suis* infection.

IL-10 is a vital anti-inflammatory cytokine known for its suppressive effects on limiting the pathogenesis of inflammatory diseases (Biswas et al., 2022). Notably, lower levels of IL-10 were observed in the humanized mice (**Figure 3B**), suggesting that HLA-A11/DR1 may extend the presence of pro-inflammatory factors and interfere with IL-10 secretion during infection.

In summary, compared to murine H2, these data demonstrate the diminished capacity of human HLA-A11/DR1 to regulate inflammatory downregulation in the host.

### 3.3 IL-10 promote inflammation receding and bacterial clear during infection

**Figure 5:**
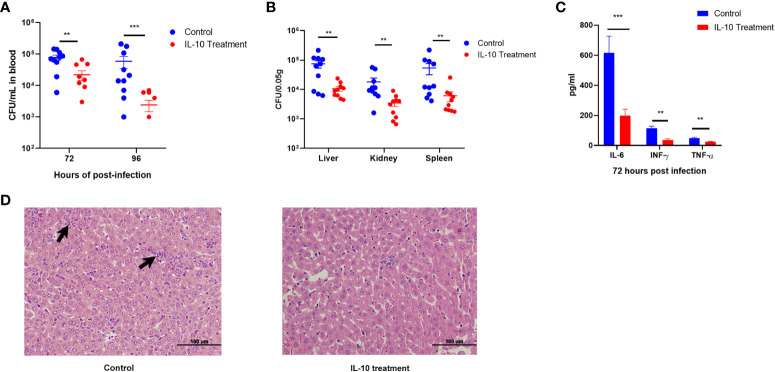
IL-10 facilitates inflammatory resolution and enhances bacterial clearance in the late stage of infection in HLA-A11/DR1 mice. Recombinant murine IL-10 or PBS was administered to HLA-A11/DR1 mice at 48 h post-infection. **(A)** Bacterial load in the bloodstream at 72 h and 96 h post-infection. **(B)** Bacterial load in multiple organs at 72 h determined through plate counting. **(C)** Levels of IL-6, IFN-γ, and TNF-α in plasma, and **(D)** histopathological change in liver at 72 h post-infection were monitored. Inflammatory cell infiltration is indicated by a “black arrow”. Data are presented as the mean ± SEM. **P < 0.01; ***P < 0.001.

The study mentioned above reveals a coexistence of elevated bacterial loads and heightened levels of inflammatory factors in the late stage of infection in HLA-A11/DR1 mice. While the activation of neutrophils is crucial for host defense, an excessive infiltration and activation of immune cells can lead to severe tissue damage and exacerbate bacterial infections (Qin et al., 2022) The study also underscores the significance of IL-10, a cytokine known for its anti-inflammatory properties, which plays a central role in infection by regulating the immune response to pathogens and thereby safeguarding the host against excessive damage (Saraiva and O'Garra, 2010). In this study, HLA-A11/DR1 mice were subjected to IL-10 intervention at 48 h post-infection, resulting in an enhanced clearance of bacteria from the bloodstream and various organs (including the liver, kidney, and spleen) at 72 h post-infection (**Figures 5A, B**). Simultaneously, IL-10 treatment in the late stage of infection effectively mitigated the production of pro-inflammatory factors such as IL-6, INF-γ, and TNF-α at 72 h post-infection, which are consequences of the inflammatory response (**Figure 5C**). Furthermore, it curtailed the tissue damage caused by inflammatory cells (**Figure 5D**). In sum, the reduced secretion of IL-10 in HLA-A11/DR1 mice leads to persistent inflammatory damage and tissue injury in the late stage of infection, promoting bacterial proliferation in both tissue and blood.

### 3.4 Humanized mice have a profound impact on immune cell activation and differentiation

**Figure 6:**
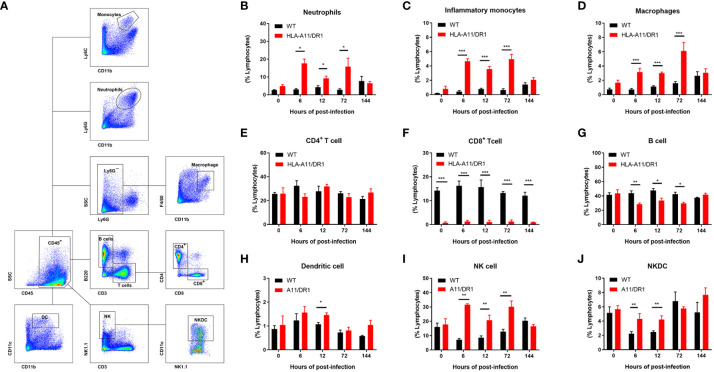
Human HLA-A11/DR1 impacts immune cell regulation post-infection. Multiparametric flow cytometric analysis quantified leukocyte recruitment in the spleen of WT and HLA-A11/DR1 mice at various time points post-infection with 1 × 10^8^ CFU of *S. suis*. **(A)** Gating strategy employed for identifying leukocyte populations in the spleen. **(B-J)** The percentage of different invading leukocytes in WT and HLA-A11/DR1 mice (n=3-5). Data are represented as mean ± SEM. *P < 0.05; **P < 0.01; ***P < 0.001.

To enhance our understanding of Humanized HLA-A11/DR1 Tg mice, immune cell response and regulation during *S. suis* infection was monitored comprehensively. Following *S. suis* inoculation in WT and HLA-A11/DR1 mice, splenocytes were harvested at specified time points (0 h, 6 h, 12 h, 72 h and 144 h). Flow cytometry was employed to define the percentage of leukocytes, and the gating strategy is shown in **Figure 6A**. Increased leukocyte recruitment (including neutrophils, monocytes and macrophages) in HLA-A11/DR1 mice at 6 h, 12 h and 72 h post-infection (**Figures 6B–D**). Those increased cells explained the lower bacterial load in the HLA-A11/DR1 mice at 6 h post-infection, and an impaired resolution during the late stage of infection (**Figures 1A**, **2**). This observation also corresponded to elevated levels of cytokines (TNF-α, INF-γ, and IL-6) quantified in cell-free biological fluids (**Figures 3E, H, I**). While these cells serve as sources of proinflammatory mediators and play crucial roles during bacterial infection, the growth of these cells was not as pronounced as in the WT group. These results suggest that human HLA-A11/DR1 molecules induce a more intense inflammatory reaction than murine H2 molecules in response to *S. suis*.

In addition to the granulocytes involved in innate immunity, a diverse array of antigen-presenting cells and lymphocytes involved in adaptive immunity were also intensively monitored. Analysis of CD4^+^ T-lymphocyte numbers revealed no significant differences between the two genotypes of mice strains (**Figure 6E**). Despite a notable reduction in CD8^+^ T cells in HLA-A11/DR1 mice, consistent with earlier studies on Humanized MHC Tg mice (Li et al., 2019), the overall trend of change remained the same between the two groups throughout the entire infection stage (**Figure 6F**). It is worth noting a substantial decrease in B lymphocytes (B cells) in post-infection HLA-A11/DR1 mice (**Figure 6G**), which suggests a more rapid and active acquired immune response. Additionally, heightened levels of dendritic cell (DC) were observed at 12 h post-infection, along with increased activation of Natural killer (NK) and Natural killer dendritic cell (NKDC) at 6 h and 12 h in the HLA-A11/DR group (**Figures 6H–J**).

In summary, these findings demonstrate that HLA-A11/DR1 promotes the recruitment of pro-inflammatory cells and hinders timely resolution. HLA Tg mice, expressing HLA-A11/DR1 monochain molecules in an H-2 class I/II^(-)^ context, display an enhanced capacity to develop long-lasting inflammatory responses compared to WT mice. Resolution dysfunction following *S. suis* sepsis is exacerbated in transgenic HLA-A11/DR mice.

### 3.5 HLA has no immediate influence on the bactericidal abilities of granulocytes *in vitro*

**Figure 7:**
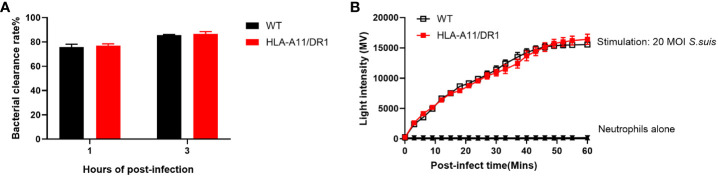
Impact of human HLA-A11/DR1 on bactericidal abilities *in vitro*. WT or HLA-A11/DR1 mouse-derived neutrophils co-culture with live *S. suis* (MOI = 5) *in vitro*. **(A)** Bacterial clearance rates were examined in 1 h and 3 h, respectively. **(B)** ROS productions were measured by light intensity when neutrophils encountered *S. suis* (MOI = 20). Data are represented as mean ± SEM.

Neutrophils, vital immune cells of innate immunity, play essential roles in bacterial clearance and immune responses, including phagocytosis, killing, degranulation, and proinflammatory mediator production (Liew and Kubes, 2019). Notably, MHC class II is expressed in addition to MHC class I in neutrophils (Ferreira-Duarte et al., 2017). The earlier finding suggests a pro-recruitment propensity of HLA-A11/DR1 after infection, while the role of HLA-A11/DR1 in neutrophil bactericidal capacity remains unknown. To assess whether human HLA affects the bactericidal capacity of neutrophils, we examined neutrophils from both WT and Tg mice to assess bacterial clearance capacities *in vitro*. The results of bacterial clearance rate and oxidative stress properties indicated no significant differences in the bactericidal capacity of neutrophils between WT and HLA-A11/DR1 groups *in vitro* (**Figures 7A, B**). Humanized HLA-A11/DR1 molecules did not impact the bactericidal capacity of neutrophils individually but did promote neutrophil recruitment after infection.

### 3.6 HLA affects macrophage bacterial clearance by influencing mitochondrial ROS production

**Figure 8:**
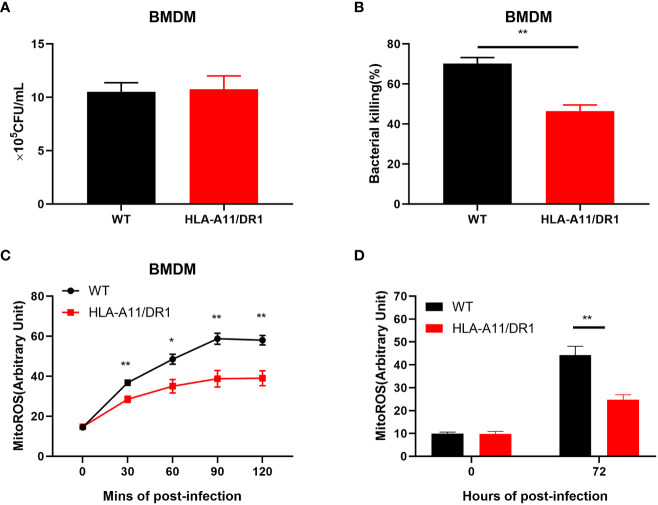
Influence of HLA-A11/DR1 on macrophage mitochondrial ROS-associated bacterial clearance. BMDM cells from WT and HLA-A11/DR1 mice were subjected to infection with *S. suis* at a MOI of 10. Subsequently, gentamicin treatment was applied 60 minutes post-infection to eliminate extracellular bacteria. The quantification of live bacteria was accomplished by plating on THY agar supplemented with ampicillin at **(A)** 1 h or **(B)** 6 h post-infection. Mitochondrial ROS production was assessed under the following conditions: **(C)** BMDM cells exposed to *S. suis* at MOI 20 at the specified time points *in vitro*, and **(D)** CD11b^+^ cells collected from WT and HLA-A11/DR1 mice during *S. suis* infection. Data are represented as mean ± SEM. *P < 0.05; **P < 0.01.

Macrophages are immune cells that play a pivotal role in bacterial clearance and the attenuation of inflammation during the late stage of infection. Consequently, we conducted comprehensive studies on their functions, both *in vitro* and *in vivo*. *In vitro* experiments, BMDM cells were used to study macrophage phagocytosis as well as bacterial killing. Bacterial counts within BMDM cells from both WT and HLA-A11/DR1 genotypes at 1 h and 6 h time points post-infection was monitored (**Figures 8A, B**). Significantly, a divergence between the two genotypes became evident at the 6 h mark, demonstrating a reduced bacterial killing function, not phagocytosis, in the HLA-A11/DR1 group compared to the WT group (**Figure 8B**). Given that mitochondrially-derived ROS play a central role in the bactericidal capabilities of macrophages (Yuan et al., 2020; Dunham-Snary et al., 2022), we conducted additional assays to measure mitochondrial ROS production for bacterial and BMDM co-culture system. The results indicate that HLA-A11/DR1 macrophages exhibited lower mitochondrial ROS production, reinforcing the observed difference between the two groups (**Figure 8C**).

To validate these findings, macrophages from both genotype mice at the late stage of infection (72 h post-infection) was collected for a mitochondrial ROS study (**Figure 8D**). Consistently, the results corroborated our *in vitro* findings, revealing reduced macrophage mitochondrial ROS production in the HLA-A11/DR1 group, which corresponded to poorer bacterial clearance when compared to the WT group. This observed reduction in mitochondrial-derived ROS production indicates that HLA-A11/DR1 status influences the bactericidal capacity of macrophages by impacting their ROS production in mitochondria.

In summation, our results collectively demonstrate that HLA-A11/DR1 exerts a considerable influence on macrophage mitochondrial ROS production in response to *S. suis*, as well as on bacterial clearance.

### 3.7 HLA-A11/DR influences the regulation of Treg cells during *Streptococcus suis* infection

**Figure 9:**
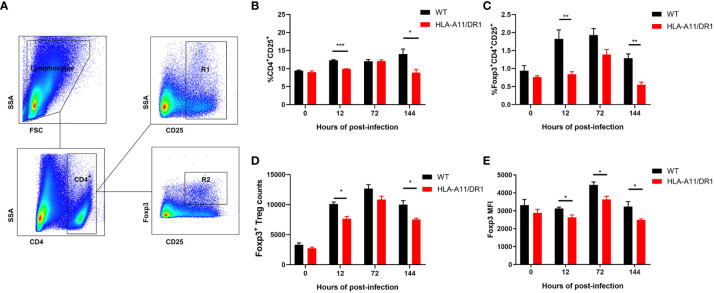
Effect of human HLA-A11/DR1 on Treg cell levels in *S. suis*-infected mice. WT and HLA-A11/DR1 mice were sacrificed at different time points (0 h,12 h, 72 h and 144 h) post-infection to obtain spleen single cell suspensions. CD4, CD25, and Foxp3 expressions were analyzed in splenic lymphocytes. **(A)** Gating strategy employed for identifying splenic leukocyte populations. CD25^+^ T cells gated by CD4 were divided into CD4^+^CD25^+^ (R1) and CD4^+^CD25^+^Foxp3^+^ (R2) populations. **(B)** Percentage of CD4^+^CD25^+^ cells and **(C)** CD4^+^CD25^+^Foxp3^+^ T cells in CD4^+^ T cell subpopulation were assessed. **(D)** Count of Foxp3^+^ Treg cells in per 100,0000 lymphocyte and **(E)** MFI of CD4^+^CD25^+^Foxp3^+^ T cells were determined by FACS. Data are represented as mean ± SEM. *P < 0.05; **P < 0.01 ***P < 0.001.

The previous study revealed robust immune activation and inflammatory upregulation in HLA-A11/DR1 mice. Regulatory T cells (Tregs), recognized as key regulators of immune homeostasis, are phenotypically defined as CD4^+^ CD25^+^ lymphocyte subsets. We closely monitored the temporal dynamics of Tregs in the spleen at different time points (0 h, 12 h, 72 h, and 144 h) post-infection. Forward and side scatter properties were used to gate splenic lymphocytes, followed by analysis of the percentage of CD4^+^ CD25^+^ Tregs and CD4^+^ CD25^+^ Foxp3^+^ Tregs within the CD4^+^ T cell subpopulation (**Figure 9A**). At 12 h and 144 h post-infection, a higher percentage of CD4^+^ CD25^+^ and CD4^+^ CD25^+^ Foxp3 cells were observed in the HLA-A11/DR1 Tg group (**Figures 9B, C**). Since Foxp3 serves as a critical regulatory gene for Tregs’ function and development (Chen et al., 2018), the count of CD4^+^ CD25^+^ Foxp3^+^ Treg cells and mean fluorescence intensity (MFI) were also analyzed. A distinct depression in the count of Foxp3^+^ Tregs (per 100,000 lymphocyte) and MFI was noted in HLA-A11/DR1 mice at 12 h and 144 h (**Figures 9D, E**).

Overall, these results indicate that HLA influences the regulation of Treg cells during infection, potentially contributing to impaired inflammatory regression and an extended course of infection in *S. suis* infection.

## 4 Discussion

HLA genes play a pivotal role in the host’s response to foreign pathogens (Langton et al., 2021). This study aims to elucidate the divergent MHC-associated immune responses in mice and humans during *S. suis* infection. Comparative analysis between murine H2 and humanized Tg mice reveals that HLA-A11/DR1 prolongs bacteremia and exacerbates pro-inflammatory cytokine production, underscoring its role in impeding timely resolution and heightening susceptibility to *S. suis*. The diminished infiltration of Treg cells and reduced IL-10 levels in HLA-A11/DR1 mice likely underlie this disparity.

The significance of MHC molecules in viral infections and autoimmune diseases is well-documented, with humanized mice traditionally linked to viral rather than bacterial infections (Zottnick et al., 2020; Medetgul-Ernar and Davis, 2022). This study marks the inaugural comparison of *S. suis* infections in humanized and wild-type mice, emphasizing the pivotal role of HLA-A11/DR1 in evaluating treatment efficacy and uncovering pathogenic mechanisms.

At the population level, the diversity of human HLA molecules likely maximizes the probability of immune responses against emerging infections and survival (Dendrou et al., 2018). Genetic variations in resistance or susceptibility to different endemic infectious agents have been observed across various human populations (Sanchez-Mazas et al., 2012). Studies highlight the reduced susceptibility to severe systemic diseases induced by group A streptococcal infections in patients with the DRB11501/DQB10602 haplotype (Kotb et al., 2002).

It is noteworthy that the highly virulent strain of S. suis has been associated with outbreaks in humans, primarily originating from infected pigs. Consequently, the polymorphism within the swine leukocyte antigen (SLA) system may represent a crucial determinant. Multiple studies have demonstrated the significant influence of SLA on various traits, including bodyweight and reproductive characteristics (Imaeda et al., 2018; Matsubara et al., 2018). Furthermore, a higher number of SLA class I haplotypes, with 28 known and 4 potential haplotypes, have been observed in the commercial pig population of Thailand, in contrast to 19 in the Danish population and 23 in the US population (Techakriengkrai et al., 2021). The dominant haplotype within these pig populations also exhibits variation (Pedersen et al., 2014). These disparities in SLA haplotypes within commercial pig populations may potentially contribute to the occurrence of swine infections in distinct geographic regions. Consequently, comprehensive knowledge of prevalent SLA alleles within the population is not only imperative for genetic enhancement but also holds significance for the development of future vaccine strategies. The involvement of SLA in the susceptibility of commercial pig populations to infections is likely to remain an intriguing area of research.

In China, human *S. suis* infections are often associated with STSLS, characterized by excessive inflammation and heightened cytokine levels. IL-6 has been identified as a critical inflammatory factor in *S. suis*-induced diseases, linked to cytokine storms and tissue damage (Sanchez-Mazas et al., 2012; Hohnstein et al., 2020). This study reveals higher IL-6 levels in humanized mice at 72 h compared to WT mice. Notably, the pro-inflammatory burst induced by *S. suis* in mice usually resolves by 72 h post-infection. The elevated IL-6 levels in the late infection stage suggest impaired pro-resolution functionality in humanized mice.

A growing body of evidence underscores IL-6’s role in balancing Th17 cells and regulatory T cells, thereby inhibiting TGF-β-induced Treg differentiation (Kimura and Kishimoto, 2010; Yan et al., 2021). Other research on *S. suis* indicates that IL-10 induction reduces TNF-α and IL-6 secretion (Korn and Hiltensperger, 2021). In this context, *S. suis* stimulation triggers high levels of IFN-γ and low levels of IL-10 in the humanized mouse model. Similar findings are apparent in another study involving HLA-A11/DR1 mice assessing tuberculosis (TB) vaccine peptides-based vaccines. This study demonstrates the humanized mouse model’s superior immune protection post-TB vaccines compared to WT mice, emphasizing the significance of animal model selection for vaccine evaluation (Gong et al., 2021).

In recent years, HLA Tg humanized mouse models have proven invaluable for studying HLA-related mechanisms in vaccine development. The emergence of the HLA-B27 Tg rat model has contributed to novel theories about HLA-B27 pathogenicity, encompassing innate immunity dysregulation and microbiota influence on diseases (Breban et al., 2021). For over a decade, HLA Tg mice have served as prototypical models for T cell-mediated autoimmunity and reproducing idiosyncratic drug toxicity. Humanized mice offer promise in reconstituting immune responses during infections within a human-like context. The use of HLA-A2-Tg, NOD-scid-IL-2γ receptor-knockout mice in reconstituting human hematopoiesis has enhanced the understanding of Ebola virus and Reston virus pathogenesis (Escudero-Perez et al., 2019). These Tg models have advanced our comprehension of the role of human molecules in disease predisposition and induction.

On the topic of Tregs, the TCR consists of an α-β heterodimer limited to antigens presented by MHC-II (Eggenhuizen et al., 2020). Beyond conferring antigen-specificity, the TCR’s affinity for a given peptide–MHC complex governs the potency of the Treg response. Upon Treg activation, it can broadly suppress through direct antigen-specific immune tolerance via cell-to-cell crosstalk mechanisms or bystander suppression through the secretion of inhibitory cytokines (such as IL-10, IL-35, and TGF-β), which inhibit nearby cell responses (Guo et al., 2020).

An intriguing phenomenon was observed in the course of this study, where a simultaneous presence of high bacterial loads and heightened inflammation was noted in transgenic mice at the 72 h post-infection. Neutrophils, which commonly serve as bacterial scavengers in microbial infectious diseases, played a central role in this context. It is well-established that inflammation, coupled with the hyperactivation of neutrophils, can result in tissue damage, thereby facilitating the proliferation of bacteria within the affected tissues. Notably, the present investigation revealed that IL-10 treatment in HLA-A11 mice led to a reduction in bacterial load, offering support for this notion. The recruitment of neutrophils serves as a hallmark of the activation of the inflammatory response. While the activation of neutrophils is pivotal for host defense, an excessive infiltration of these immune cells can inflict severe tissue damage and exacerbate bacterial infections, as previously noted (Lehman and Segal, 2020). The activation of neutrophils can either induce or exacerbate tissue injury, underscoring the importance of achieving a balance between activation and the subsequent resolution of inflammation once the infection has been successfully cleared. In the study by Aguilera and Lenz, their research on model pulmonary infections and co-infections demonstrates the indispensable role of immune effector recruitment in eliminating bacteria and virus-infected cells, although it should be noted that the inflammatory cytokines generated in the process can alter the pulmonary environment in a manner conducive to increased pathogen replication (Aguilera and Lenz, 2020). In our current investigation, it was observed that HLA-A11/DR macrophages exhibited diminished bacterial clearance capability during *S. suis* infection, possibly attributed to a reduced production of ROS in phagocyte (especially mitochondria-derived ROS). In contrast, IL-10 was found to enhance the bacterial clearance potential of macrophages, thereby promoting the regression of inflammation. These findings align with a study on *Acinetobacter baumannii*, where IL-10-deficient mice displayed elevated mortality rates, an excess production of pro-inflammatory cytokines in the lungs, and increased bacterial burdens following infection (Kang et al., 2020). Notably, IL-10 demonstrated its capacity to shield mice from lung infections caused by *Acinetobacter baumannii* by regulating STAT3-mediated MARCO expression in macrophages. Furthermore, it is worth mentioning that certain bacterial pathogens, such as *Klebsiella pneumoniae*, strain ST258 can evade the initial defenses of innate immunity by reducing the production of ROS by polymorphonuclear neutrophils. This evasion strategy can lead to the co-occurrence of heightened inflammation and increased bacterial loads within the host (Castillo et al., 2019). Overall, with regard to the precise molecular mechanisms underlying the coexistence of elevated inflammation and heightened bacterial loads, further extensive research is imperative to substantiate these findings.

Our findings unveil that humanized HLA-A11/DR1 mice experience prolonged infection, impaired resolution, and increased bacterial load during *S. suis* infection. The attenuated Treg activation and lower IL-10 levels in HLA-A11/DR1 mice likely contribute to these outcomes. HLA-A11/DR1 Tg mice exhibit potential as suitable animal models for infection model development, therapeutic efficacy assessment, and exploration of infection mechanisms.

## Data availability statement

The original contributions presented in the study are included in the article/supplementary material. Further inquiries can be directed to the corresponding authors.

## Ethics statement

The animal study was approved by the Institutional Animal Care and Use Committee of the Academy of Military Medical Sciences, China. The study was conducted in accordance with the local legislation and institutional requirements.

## Author contributions

CN: Formal Analysis, Methodology, Writing – original draft, Investigation. YH: Writing – original draft, Investigation. YW: Writing – original draft, Investigation. TM: Writing – original draft, Formal Analysis. DS: Writing – original draft, Formal Analysis. YX: Writing – original draft, Investigation. WC: Funding acquisition, Supervision, Writing – review & editing. SG: Funding acquisition, Supervision, Writing – review & editing, Project administration.
